# Integrating testing for chronic strongyloidiasis within the Indigenous adult preventive health assessment system in endemic communities in the Northern Territory, Australia: An intervention study

**DOI:** 10.1371/journal.pntd.0008232

**Published:** 2020-05-13

**Authors:** Wendy A. Page, Jenni A. Judd, David J. MacLaren, Petra Buettner

**Affiliations:** 1 Miwatj Health Aboriginal Corporation, Nhulunbuy, Northern Territory, Australia; 2 College of Medicine and Dentistry, Division of Tropical Health and Medicine, James Cook University, Cairns, Queensland, Australia; 3 School of Health, Medical and Applied Sciences, Division of Higher Education, Central Queensland University, Bundaberg, Queensland, Australia; 4 Centre for Indigenous Health Equity Research, Central Queensland University, Bundaberg, Queensland, Australia; 5 Centre for Health Systems Strengthening, James Cook University, Townsville, Queensland, Australia; 6 Centre for Chronic Disease Prevention, James Cook University, Cairns, Queensland, Australia; 7 Tropical Health Solutions, Queensland, Australia; Instituto de Investigaciones en Enfrmedades Tropicales, Universidad Nacional de Salta, ARGENTINA

## Abstract

**Background:**

The life-threatening clinical manifestations of strongyloidiasis are preventable with early detection and effective treatment. The aim of this study was to assess if there was an increase to the number and proportion of persons tested for chronic strongyloidiasis, as a result of integrating *Strongyloides stercoralis* serology into the existing preventive health assessment system in four Aboriginal health services in endemic communities.

**Methodology:**

A prospective, longitudinal, before-and-after intervention study was conducted in four Aboriginal health services in remote endemically infected communities in the Northern Territory, Australia, from July 2012 to December 2016. The electronic patient information and recall systems enabled the integration of *Strongyloides stercoralis* serology into the adult preventive health assessment. *Strongyloides* reports for each health service were extracted half-yearly to examine the number and proportion of persons tested for chronic strongyloidiasis during the study and to measure the effect of the intervention.

**Principal findings:**

The number and proportion of persons tested increased significantly during the study. From a total resident population of 3650 Indigenous adults over 15 years of age, 1686 persons (47.4%) were tested. The percentage of adults who had at least one serology test increased in all four health services to between 41% (446/1086) and 81.9% (172/210). Of the 1686 persons tested, 680 positive cases of chronic strongyloidiasis (40.3%) were identified.

**Conclusions/Significance:**

This population health systems intervention increased the number and proportion of persons tested for chronic strongyloidiasis in four health services in endemically infected communities. This intervention is relevant to other health services with high-risk populations.

## Author summary

Strongyloidiasis is a neglected tropical disease that is endemic in some Aboriginal communities in the Northern Territory. This study asks if the number and proportion of persons tested for chronic strongyloidiasis can be increased by incorporating a *Strongyloides* serology test into the existing routine Indigenous adult preventive health assessment system in remote endemic communities. This study demonstrated that integrating *Strongyloides* serology test within the Indigenous adult preventive health assessment system does increase the number and proportion of people tested in endemic communities. This intervention means that life-threatening clinical complications of strongyloidiasis can be prevented by early detection and treatment. Primary health care services have an important role in increased testing in this high-risk population. Primary health care clinicians incorporated chronic strongyloidiasis with other preventable chronic and infectious diseases. The sustainable population health systems-based approach successfully increased coverage by integrating testing for chronic strongyloidiasis into the adult preventive health assessment in health services in remote Indigenous Australian endemic communities, utilising the electronic health record system. The *Strongyloides* report developed to measure the change in clinical practice would be replicable in other health services with high risk populations.

## Introduction

Strongyloidiasis is described as “the most neglected of neglected tropical diseases” [1] with an estimated 370 million people infected worldwide [2]. The disease is caused by the microscopic soil-transmitted helminth *Strongyloides stercoralis*. The unique autoinfective cycle whereby offspring from the parasitic female adult reinfect the host distinguishes *Strongyloides stercoralis* from all other soil-transmitted helminths that infect humans [3, 4]. The autoinfective cycle ensures that persons remain infected for life, unless effectively treated [5]. *Strongyloides stercoralis* persists indefinitely in the infected person, whereas the external life-cycle of this helminth is limited to a few weeks [6–8]. When environmental health infrastructure prevents transmission of soil-transmitted helminths, persons infected with *Strongyloides stercoralis* still have a life-time risk of a life-threatening event. This autoinfective cycle of *Strongyloides stercoralis* requires a different clinical approach to diagnosis and treatment compared to other soil transmitted helminths.

Chronic strongyloidiasis with intermittent, non-specific symptoms is unlikely to be diagnosed, unless considered by the clinician [9–11]. The autoinfective larvae can travel randomly to any organ of the body and can transport enteric bacteria that can cause septicaemia or gram-negative meningitis [12–14]. Hyperinfection or disseminated strongyloidiasis occurs when the autoinfective cycle is amplified and the number of worms increases out of control, resulting in a high fatality rate of up to 87% [1, 15]. Septicaemia, meningitis and multiple end-organ failure are sufficient as an end-of-life diagnosis, with the underlying cause remaining undiagnosed [16].

An analysis of available published case reports on strongyloidiasis from 1876 to 2002 showed most deaths were due to strongyloidiasis being undiagnosed and untreated prior to death; being diagnosed but ineffectively treated; or immunosuppressant therapy being given to patients from high-risk groups without testing for strongyloidiasis [17]. International recommendations are to test and treat all infected persons, including the asymptomatic persons, as the life-threatening presentations are unpredictable and preventable [18–20]. Because of the risk of a life-threatening event, a system to increase testing of persons at risk is needed.

In Australia, there are endemic hot spots in many remote Indigenous communities, in the wet tropics and the deserts [11, 16, 21–23], with hyperendemic rates of strongyloidiasis of 21% to 60% in some locations [24–27]. *Strongyloides* serology has proven useful for measuring prevalence, identifying positive cases that require treatment, and measuring the effectiveness of treatment as serology declines to negative [26, 28, 29].

Chronic strongyloidiasis is diagnosed using *Strongyloides stercoralis* Immunoglobulin G (IgG) enzyme-linked immunosorbent assay (ELISA) serology which is a sensitive (92.3%) and specific (97.4%) test [18, 30, 31] well suited to the primary health care setting [9, 25, 26, 32]. Immunoglobulin G (IgG) is a measure of immune response to the presence of *Strongyloides stercoralis* rather than an indication of the number of worms. The opportune time to use serology to identify patients requiring treatment is during the chronic phase which lasts for decades. Serology is less sensitive in acute strongyloidiasis due to a time lag, typically of several weeks, between initial infection and a detectable immune response. Serology is also less sensitive in disseminated strongyloidiasis when the immunocompromised person may not be able to mount an immune response [9, 33, 34].

The goal of treatment for persons infected with *Strongyloides stercoralis* is eradication of all parasitic worms, as even a single parthenogenic parasitic female can re-establish an infection. Ivermectin is an effective first line treatment with an estimated cure rate of 85% [16, 26, 27, 35–37]. Best practice recommends diagnosis, treatment and follow-up serology to measure the effectiveness of treatment. A decline to negative serology may take 6 to 12 months and is an indication of cure. [25, 26, 28, 32, 38]. Re-treatment may be required.

Australian studies have provided evidence for this best practice in primary health care for refugees and immigrants in a non-endemic environment [32, 39] as well as for endemically-infected Indigenous communities [25–27, 40]. Refugees and immigrants who have had prior exposure in endemic countries are considered high-risk populations with prevalence rates of 2% to 42%, when tested several years after arrival in Australia [32, 39, 41–47]. Screening of refugees entering Australia as recommended by the Australasian Society of Infectious Diseases [47–49] now includes *Strongyloides* serology. The refugee health assessment is intended to increase the identification of positive cases needing treatment to prevent complications and prevent transmission [47]. However, screening of high-risk Indigenous populations living in endemically infected communities has not yet been specifically recommended by health policy makers in Australia [16, 17, 50, 51].

The preventive health assessment for Aboriginal and Torres Strait Islander people is a population health strategy implemented in primary health care services in Australia, to reduce health inequity between Indigenous and non-Indigenous Australians [52–54]. This national strategy aims to encourage early detection, diagnosis and management for common and treatable conditions that cause morbidity and early mortality in Indigenous communities [55]. In the Northern Territory, the Aboriginal adult preventive health assessment includes serological testing for chronic kidney disease, diabetes, chronic hepatitis B and sexually transmitted diseases. This assessment referred to locally as the adult health check (AHC) provides an opportunity to test for chronic strongyloidiasis in conjunction with other preventable chronic conditions [52, 54]. In this study, the term Indigenous is used respectfully to represent Australian Aboriginal and Torres Strait Islander people.

The aim of the study was to assess if there was an increase in the number of persons tested for chronic strongyloidiasis, as a result of integrating *Strongyloidiasis stercoralis* serology into the adult preventive health assessment system in four Aboriginal health services in endemic communities.

## Methods

A prospective, longitudinal, before-and-after design intervention study was conducted in four Aboriginal health services in the Northern Territory from July 2012 to December 2016. This study aimed to ascertain if the change in coverage (persons tested at least once as proportion of clinic population) was attributable to the population health systems intervention.

Key measurements were: (1) The number and proportion of resident adults tested at least once for chronic strongyloidiasis in four primary health care services in remote Indigenous communities; (2) If an increase in the number and proportion of resident adults tested at least once for chronic strongyloidiasis was attributable to the intervention; and (3) The number and proportion of resident adults who tested positive at least once as a proportion of those tested for each clinic and overall during the study.

### Participants and setting

Four health services (Clinics A, B, C, and D) participated in this study. All four clinics are located in remote Indigenous communities, in the Northern Territory, Australia where strongyloidiasis is endemic. All four clinics operated under the auspices of an Aboriginal Community Controlled Health Organisation. These are primary health care services, controlled by, and answerable to the local Aboriginal community, that deliver holistic, comprehensive, and culturally appropriate health care to the communities that they serve. The Aboriginal populations that were part of this study are aware of strongyloidiasis through community education and local radio programs in their primary local language of Yolngu Matha. The study was in response to community members advocating for a *Strongyloides* control program.

Aboriginal health services in the Northern Territory deliver health care to populations ranging from less than 100 to 3000 residents [52]. The population of the participating Aboriginal communities serviced by these clinics varied from 215 to 2067 residents [56]. Each Aboriginal health service in the Northern Territory uses an electronic patient information and recall system for collecting and storing all clinical information. Reports such as the half-yearly Northern Territory Aboriginal health key performance indicators (NTAHKPI) reports can be extracted from the electronic health record system in each clinic to facilitate population health planning by health care staff [52].

For this study, the selection criteria were the same as used for Indigenous AHC reports. The inclusion criteria were: Indigenous clients when they were 15 years and older, and who were classified as a current resident by the local community health service. A current resident was defined as a client of a health service who usually resided in that community or was someone who had recently moved to the community and intended to stay there. In this real-world setting, the current resident adult populations were dynamic with fluctuations throughout the 4.5-years study. Patients who deceased were excluded from the assessments after their date of demise. The exclusion criteria for the study were transient and past clients who were designated as non-residents. Non-Indigenous clients were also excluded from the study.

### The electronic patient information and recall system

All four participating clinics in this study used the Communicare patient information and recall system. In this study, Clinics A and B shared the same Communicare database, whereas Clinics C and D had separate Communicare databases.

### The population health intervention

*Strongyloides* serology was added to the routine adult health check (AHC) investigations. As part of an AHC, clinicians were prompted to request a test for strongyloidiasis. *Strongyloides* serology test results were imported electronically into the Communicare results in-tray via Health Level Seven (HL7) format from the pathology laboratory. A doctor checked the results in the electronic in-tray and generated a recall for abnormal results. Serology results were classified as positive or negative. Patients with a positive result were recalled for treatment with ivermectin 200mcg/kg, with a repeat dose after two weeks. Follow-up serology after six months was recommended to assess the effectiveness of therapy.

The Logical Observation Identifiers Names and Codes (LOINC) database was used to import a copy of results for each patient into the qualifier file. When a clinician consulted a patient, the latest qualifiers (e.g., blood pressure, weight, and serology tests for cholesterol, diabetes, renal disease, and *Strongyloides*) were available on the main summary screen to inform clinical action.

A *Strongyloides* report developed for this study extracted data from the qualifier file using the following search criteria: (1) Time frame: start date: 1^st^ July 2012. The initial end date was at 31^st^ December 2012, then at half-yearly intervals until 31^st^ December 2016. (2) Biographics: cultural group “Aboriginal” “Torres Strait Islander” “Aboriginal and Torres Strait Islander”; age “15 years and over”; residential status “current”; clinic location “clinic name”. (3) Qualifiers: most recent *Strongyloides* result and date of the test.

The internal clinic reports listed patients who were tested and those who were not tested. These reports provided the patient’s most recent test result and date of the test, further separating test results into positive and negative. The percentages for coverage (persons tested/persons tested plus not tested) and prevalence (persons positive/persons tested) were calculated and added at the end of the internal report. Each of the four participating clinics could generate their own internal *Strongyloides* reports with identified data that were available for clinical audit, feedback and planning purposes. The external de-identified half-yearly *Strongyloides* reports tracked the changes in point coverage of persons tested at least once versus persons not tested over the duration of study for each clinic based on the current adult residents at the time of data extraction.

The *Strongyloides* serology test was provided by Western Diagnostic Pathology in Perth, Western Australia using the commercial IVD enzyme-linked immunosorbent assay (ELISA) (DRG Instruments GmbH, Marburg, Germany). This assay detects anti-*Strongyloides* IgG antibodies using the *Strongyloides stercoralis* L3 filariform larval antigens. The optical density (OD) cutoff value for this test reported less than 0.2 as negative and an OD of 0.2 or greater as positive with a sensitivity of 92.3% and specificity of 97.4% [30]. This new test was introduced in all clinics from July 2012 replacing the *Strongyloides ratti* serology test. Data were therefore collected from July 2012 onwards.

### Comparators

As a before-and-after study, each clinic compared to itself. The intervention was implemented in Clinics A, B and C from July 2012 on request of clinicians who did not wish to delay the population health intervention. For Clinic D, the start of the intervention was delayed until January 2015, enabling five baseline assessments for comparison before the population health intervention commenced in Clinic D. The baseline assessments measured the usual practice of clinicians requesting a test if the patient had clinical symptoms, unexplained eosinophilia or the patient was to be immunosuppressed for other conditions [9, 18].

### Data collection

*Strongyloides* reports for all resident Indigenous patients aged 15 years and older were extracted and de-identified for analysis and included age, sex, persons tested for strongyloidiasis (yes, no). Further information on individual test results (positive, negative and OD value) and date of the test was also provided. At the end date of each *Strongyloides* report, only the most recent test result was included during the period selected. Thus, the person was counted only once in each clinic, although their testing status could change in a subsequent report. Reports were extracted nine times with a start date of 1^st^ July 2012 and with end dates at 31^st^ December 2012, 30^th^ June 2013, 31^st^ December 2013, 30^th^ June 2014, 31^st^ December 2014, 30^th^ June 2015, 31^st^ December 2015, 30^th^ June 2016, and the last report at 31^st^ December 2016.

Prior to the study, a sample size calculation showed that 94 persons tested per clinic were required to detect a 20% increase in persons tested with statistical confidence (alpha 0.05) and a power of 80% assuming 50% baseline coverage rates (worst case scenario for sample size calculation).

### Outcome measures

(1) Period coverage was the number of persons tested for strongyloidiasis at least once between July 2012 and December 2016 divided by the total number of resident population 15 years and older during the entire study period. (2) Point coverage was the number of persons tested at least once for strongyloidiasis divided by the number of current resident population aged 15 years and older at the end of each half-yearly assessment for each clinic. An activity report of persons tested newly during each half-yearly interval was conducted. (3) Period prevalence was the number of persons who tested positive for strongyloidiasis at least once between July 2012 and December 2016 divided by the number of persons tested during the entire study period.

### Statistical analysis

The analysis was conducted using Stata release 12.1 (Stata 2011). Categorical data were described using absolute and relative frequencies. Age of a patient was defined as age at date of first record of test result or last date of period of observation when first recorded. Age was the only numerical variable. The distribution of age was skewed, and it was therefore described using median values, inter-quartile ranges (IQR), and ranges.

Coverage measuring persons tested at least once for strongyloidiasis as proportion of population was calculated and presented with exact binomial 95% confidence intervals (95% CI). Point coverage of persons tested at least once was calculated at each half-yearly assessment, stratified by clinic and are presented with exact binomial 95% confidence intervals. An activity measure of persons tested newly at each half-yearly assessment was calculated for each clinic and are presented with exact binomial 95% CIs.

Exact binomial 95%CIs for period coverage and period prevalence were cluster adjusted assuming the clinics as primary sampling units. A sensitivity analysis was conducted calculating period prevalence of strongyloidiasis assuming that all patients without a serology test were negative.

For each clinic and overall, period coverage rates of persons tested at least once and period prevalence of persons testing positive at least once were compared for differences in sex using exact Fisher’s tests and for differences in age using non-parametric Mann-Whitney tests. Generalised least-square random-effect logistic regression panel data analyses (xt commands in Stata) were used to assess associations between persons tested newly each half year and time (half-yearly from July 2012 to December 2016), sex and age stratified by clinic. Results were reported as coefficients and 95% CI. P-values less than 0.05 were considered statistically significant.

### Ethics approval

Ethics approval was gained from the Human Research Ethics Committee of the Northern Territory Department of Health and Menzies School of Health Research (HREC Reference Number 2012–1868) and James Cook University (HREC Approval number H4953). The Board of Miwatj Health Aboriginal Corporation, the owners of the clinical databases with responsibility for deciding what research is appropriate and acceptable, provided written approval for this study. Individual adult patients provided oral consent for a preventive health assessment that was documented in their electronic health record. All data extracted for this study was de-identified.

## Results

### Basic characteristics of resident populations aged 15 years and older

Between July 2012 and December 2016, a total of 210 persons were recorded in Clinic A, 540 persons in Clinic B, 1086 persons in Clinic C and 1724 persons in Clinic D. Overall, 50.5% of 3560 patients were female and the median age was 30 years (range 15 to 80 years) (Table 1).

**Table 1:** Basic characteristics, period coverage of persons tested, and period prevalence of strongyloidiasis recorded for all persons in resident population aged 15 years and over by four clinics in remote locations of the Northern Territory, Australia, between July 2012 and December 2016. Number of participants counts any person recorded as a resident adult client at a clinic during the study.

	Community				
	Clinic A	Clinic B	Clinic C	Clinic D	Total
Number of participants	210	540	1086	1724	3560
N (%) Female	107 (51.0%)	293 (54.3%)	535 (49.3%)	863 (50.1%)	1798 (50.5%)
Median age (IQR) *; range [years]	31 (20, 41); range 15 to 74	31 (21, 44); range 15 to 75	30 (20, 42); range 15 to 80	29 (19, 41); range 15 to 80	30 (20, 42); range 15 to 80
N (%) with serology test at least once between 2012 and 2016; 95% CI^	172 (81.9%);76.0 to 86.9	330 (61.1%);56.9 to 65.3	446 (41.1%);38.1 to 44.1	738 (42.8%);40.5 to 45.2	1686 (47.4%);32.2 to 62.5
N (%) positive for strongyloidiasis at least once between 2012 and 2016; 95% CI^	88 (51.2% of 172); 43.4 to 58.9	126 (38.2% of 330); 32.9 to 43.7	214 (48.0% of 446); 43.3 to 52.7	252 (34.1% of 738); 30.7 to 37.7	680 (40.3% of 1686); 27.1 to 53.5
*Assuming patients not tested were negative*^^N(%) positive for strongyloidiasis at least once between 2012 and 2016; 95% CI^	88 (41.9% of 210); 35.2 to 48.9	126 (23.3% of 540); 19.8 to 27.1	214 (19.7% of 1086); 17.4 to 22.2	252 (14.6% of 1724); 13.0 to 16.4	680 (19.1% of 3560); 9.4 to 28.8

^95% CI = 95% exact binomial confidence intervals

*IQR = inter-quartile range

^^Sensitivity analysis assumed that all patients without a serology test had a negative test result.

### Coverage: Persons tested for strongyloidiasis at least once as proportion of resident population

Overall, 1686 persons (47.4%; 95% CI 32.2 to 62.5; n = 3560) were tested for strongyloidiasis at least once between July 2012 and December 2016 (Table 1). Period coverage varied between 41.1% in Clinic C and 81.9% in Clinic A (Table 1). In Clinic C, more females compared to males were tested for strongyloidiasis (p = 0.003). Bivariate statistical tests detected no further significant sex differences of period coverage for the other three clinics. In all four clinics, median ages of people who were tested for strongyloidiasis at least once were significantly higher compared to people who were not tested (Clinic A: 32.5 vs 22.5 years, p = 0.002; Clinic B: 34 vs 27 years, p<0.001; Clinic C: 35 vs 28 years, p<0.001; Clinic D: 34 vs 25 years, p<0.001).

**Fig 1:**
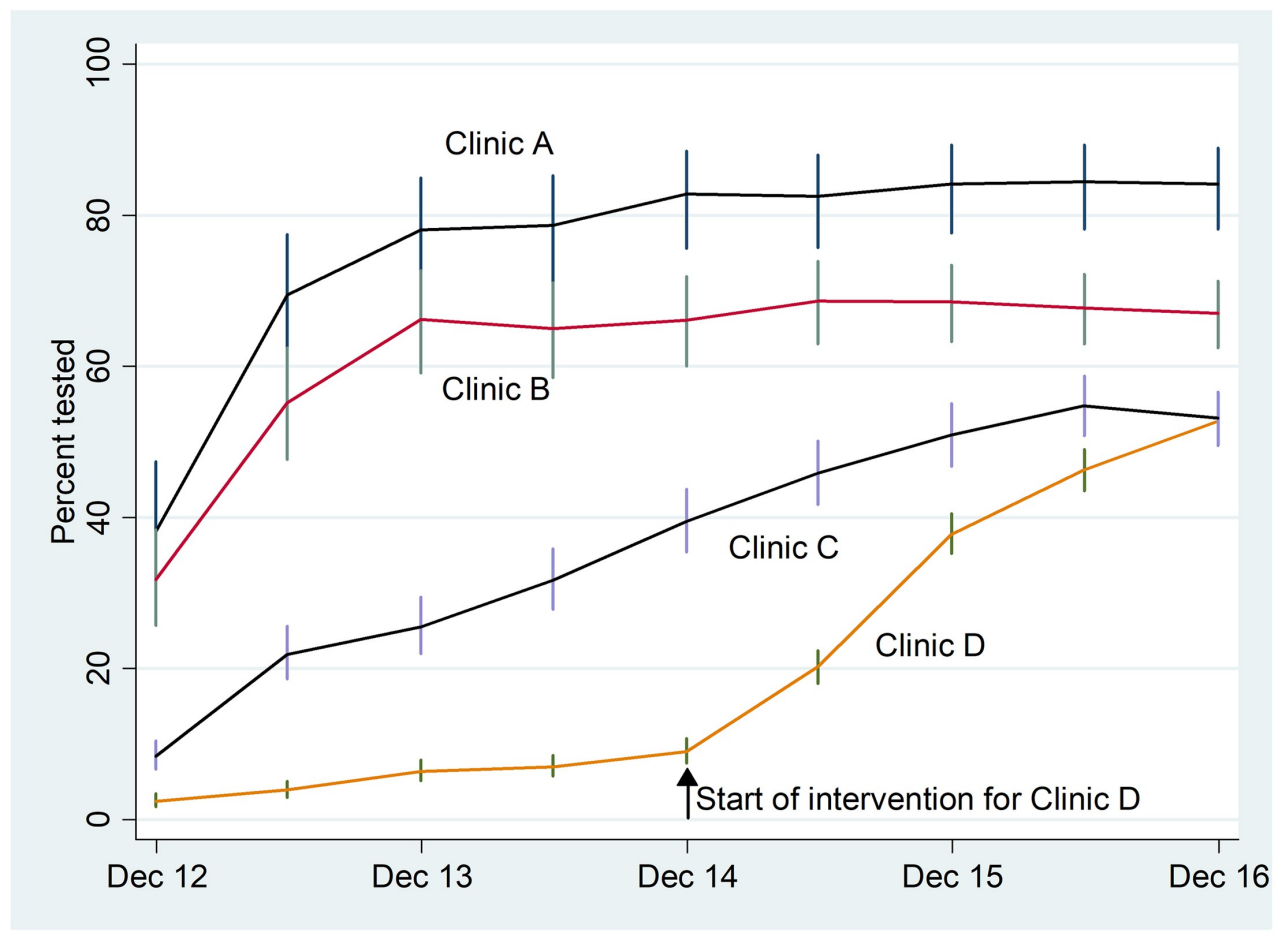
*Strongyloides* point coverage reports extracted at half-yearly intervals (cumulative over the entire 4.5 years study period, starting in July 2012; 95% confidence intervals) of persons tested at least once for strongyloidiasis as recorded for the current resident population aged 15 years and over at the time of data extraction by four clinics in remote locations of the Northern Territory, Australia, between July 2012 and December 2016 (S1 Table).

*Strongyloides* reports on point coverage at half-yearly intervals (cumulative over the entire 4.5 year study period, starting from July 2012) showed the changes in coverage with time for each clinic (S1 Table; Fig 1). In December 2016, the percentage of resident persons tested for strongyloidiasis at least once in the previous 4.5 years study period ranged between 52.7% for Clinic D and 84.1% for Clinic A. The intervention began in July 2012 for Clinics A, B, and C. The intervention began in January 2015 in Clinic D (S1 Table; Fig 1).

Result of multivariable logistic regression panel data analyses showed that the number of serological tests conducted every half-year (based on newly tested persons in each half yearly time interval) increased statistically significantly with time overall and in all four clinics separately (p<0.001, respectively) (S2 Table, Table 2). Multivariable analyses further indicated that compared to females, male patients were less likely to be tested overall (p<0.001); in Clinic C (p<0.001) but not in Clinics A, B, and D. Further, the likelihood of testing increased significantly with age overall and in all four clinics separately (p<0.001, respectively) (Table 2).

**Table 2:** Result of multivariable logistic regression panel data analyses of the serology testing for strongyloidiasis as recorded for the resident population aged 15 years and over by four clinics in remote locations of the Northern Territory, Australia, between July 2012 and December 2016.

	Characteristics in model		
	Time	Male sex	Age
**Clinic A**			
Coefficient	1.9	0.2	0.3
95% CI^	1.4 to 2.6	-2.6 to 2.9	0.1 to 0.4
p-value	P<0.001	P = 0.912	P<0.001
**Clinic B**			
Coefficient	1.3	-0.5	0.2
95% CI^	1.1 to 1.5	-1.7 to 0.8	0.1 to 0.2
p-value	P<0.001	P = 0.471	P<0.001
**Clinic C**			
Coefficient	2.0	-3.0	0.3
95% CI^	1.8 to 2.2	-3.9 to -2.0	0.26 to 0.33
p-value	P<0.001	P<0.001	P<0.001
**Clinic D**			
Coefficient	3.3	0.03	0.3
95% CI^	2.9 to 3.6	-0.8 to 0.8	0.2 to 0.3
p-value	P<0.001	P = 0.948	P<0.001
**Total**			
Coefficient	2.1	-0.9	0.24
95% CI^	2.0 to 2.2	-1.4 to -0.4	0.22 to 0.26
p-value	P<0.001	P<0.001	P<0.001

^95% CI = 95% confidence interval

### Period prevalence of strongyloidiasis

Overall, 680 persons (40.3%; 95% CI 27.1 to 53.5) tested positive for strongyloidiasis at least once between 2012 and 2016 from the 1686 tested persons (Table 1). This period prevalence for strongyloidiasis varied between 34.1% in clinic D and 51.2% in clinic A (Table 1). Under the assumption that all persons who were not tested for strongyloidiasis between July 2012 and December 20016 were negative for the disease, prevalence of strongyloidiasis was 19.1% (95% CI 9.4 to 28.8; n = 3560) (Table 1).

The prevalence of strongyloidiasis was not statistically significantly different between the sexes. In clinic A, people diagnosed with strongyloidiasis were on average older (median age 35.5 compared to 29 years, p = 0.002). There was no statistically significant age difference detected in the other three clinic populations.

## Discussion

This study has documented a statistically significant increase in the number of people who were tested for chronic strongyloidiasis in each of the four participating clinics, and the increase was attributable to the intervention. The population health intervention successfully added *Strongyloides* serology tests into the adult preventive health assessment using the existing electronic patient information and recall system, in four Aboriginal health services in endemic communities in the Northern Territory. Clinicians incorporated chronic strongyloidiasis into their clinical practice as another preventable chronic and infectious diseases for early detection, treatment and management.

The *Strongyloides* reports developed for this study enabled measurement of change in coverage at regular half-yearly intervals. The reports were also internally accessible to clinicians. The intervention was implemented and demonstrated to be effective in increasing coverage (the number and proportion of persons tested at least once) in each clinic. The delay of 2.5 years in implementation for the geographically separated remote Clinic D, enabled comparison with the usual clinical practice of only testing when a clinician considered a possible diagnosis of strongyloidiasis or prior to immunosuppression [9, 18]. Thus, the prolonged baseline in Clinic D provided further evidence that the observed changes were attributable to the intervention (Fig 1).

Testing of previously untested persons continued throughout the study period, although the coverage rate appeared to have plateaued (S1 Table, Fig 1). Some apparent anomalies in the results are explained by the dynamic population in these communities. For example, in Clinic C the coverage percentage decreased from 54.8% (342/624) in June 2016 to 53.1% (412/776) in December 2016 because the population increased, despite the fact that 70 more untested persons were tested compared with 45 persons in the previous half-yearly interval (S1 Table). The coverage In Clinic A in December 2014 was 82.8% (120/145) and in December 2016 was 84.1% (164/195) appearing to have plateaued, yet an additional 44 previously untested persons were tested. The coverage in Clinic B in December 2014 was 66.1% (168/254) and in December 2016 it was 67.0% (311/464), yet an additional 143 previously untested persons were tested. Continued testing of previously untested persons is ongoing in a dynamic population, although this strategy is reliant on persons attending the health service and does not cover 100% of the population.

### Comparison with other studies

No other studies were found that measured trends in coverage rates of persons tested for strongyloidiasis through preventive health assessments. Accordingly, direct comparisons were not possible. However, measuring trends in coverage rates is a population health strategy used for continuous quality improvement, and as a planning tool in Aboriginal primary health care services in the Northern Territory. The Northern Territory Aboriginal Health key performance indicators (NTAHKPIs) were introduced in 2009 [52]. The 2016 NTAHKPIs report included a selection of data collected between 2010 to 2014. This population health reporting system facilitated improvements in the coverage of adult health checks, and other NTAHKPIs such as immunisations and cervical screening in the Northern Territory [52, 57].

Evidence supporting the successful integration of *Strongyloides* serology tests within the AHC can be shown by comparison to the NTAHKPI report on AHCs [52]. The coverage rates for strongyloidiasis testing achieved after implementing the intervention, as seen in Fig 1, were similar to the coverage rates of AHCs [52]. The smaller clinics achieved higher coverage of AHCs compared to the larger clinics, which supports the findings in this study [52]. Similarly, characteristics of residents who were tested for strongyloidiasis as part of an AHC reflect the overall characteristics of residents who received an AHC in these settings. Women and older people were more likely to receive a completed AHC [52], reflecting the results from this study showing women (in clinic C and overall) and older people at all four clinics were more likely to be tested for strongyloidiasis. Women were more likely to attend the clinic with children and older persons were more likely to attend the clinics for other health reasons and comorbidities. As AHCs are offered opportunistically when resident patients attend the clinic, gender and age groups tested for strongyloidiasis were representative of those attending the clinic, rather than of the population.

Increasing coverage and sustainability are important in control programs for Neglected Tropical Diseases (NTDs) [58]. Conway et al in 1995 advocated that control programs for strongyloidiasis in endemic communities were feasible [8]. In the northern Australian setting, four studies demonstrated benefits in endemic communities [25–27, 40]. However, these studies were of limited duration. Initial testing for chronic strongyloidiasis does not inform when or where the initial infection was acquired. An on-going population health approach is needed to identify new cases or re-infections in endemic communities. This intervention used the electronic health record system to build sustainability and increase coverage. This study provided evidence supporting the advantages of integrating this NTD into the comprehensive preventative health assessments in the remote health services in endemic communities in Australia.

### Generalisability

This same intervention was replicated four times in different clinics and found to be effective and reliable. The 4.5 years duration of the study also provided evidence that the system was sustainable, even in a setting with a high turnover of clinicians [59]. The data was extracted electronically, thus increasing the reliability of persons counted and their pathology results. The reporting tool would be transferable to other endemic communities with similar electronic patient information and recall systems.

The overall period prevalence across all 4 clinics was 40.3% with period prevalence rates ranging between 34.1% and 51.2% across clinics. These results add to the evidence of hyperendemic rates of strongyloidiasis in the Northern Territory and other hotspots across Australia [23–27]. Although prevalence varies between clinic locations, ongoing population health strategies are required to address this serious and preventable disease.

The Australian national guide to preventive health assessments for Aboriginal and Torres Strait Islander people provides recommendations within a screening, behavioural, chemoprophylaxis and environmental interventions framework for a diverse range of conditions, including those that are rare in mainstream or NTDs limited to geographic areas [53]. Strongyloidiasis is not currently in this guide. The results from this study add to the existing evidence that strongyloidiasis needs to be considered for this guide.

### Clinical relevance

This systems intervention resulted in testing 1686 persons and identifying 680 cases of chronic strongyloidiasis over the 4.5 years duration (2012–2016). This demonstrates how including *Strongyloides* testing into routine clinical practice in endemic communities can identify undetected strongyloidiasis and build on other Australian studies that showed that positive serology decreased with effective treatment in endemic communities [25–27, 40].

Test results need to be carefully considered in this population as immunocompromised patients and patients with disseminated strongyloidiasis may not generate sufficient IgG, resulting in a false negative result [9, 33]. Northern Territory hospital protocols recommend ivermectin treatment prior to immunosuppression, for all patients from highly endemic areas, irrespective of the serology results [60], acknowledging the challenges of false negative serology in immunocompromised patients. False positives from cross-reactivity are unlikely to impact the results as other parasitic diseases such as filariasis, onchocerciasis, ascariasis, and schistosomiasis that cause cross-reactivity [30] are not found in this region, and hookworm is now uncommon following the introduction of albendazole [22].

### Public health implications

Elimination of strongyloidiasis in endemic communities will not happen until environmental health conditions that facilitate transmission are addressed and the infected population are treated. Transmission continues in endemic areas where there is inadequate sanitation. Intersectoral collaboration is required to interrupt transmission at the community level, and to ensure that the most disadvantaged communities are not neglected. Adding to the challenge is the fact that there is currently no national surveillance system in Australia for the geographical mapping of this NTD to identify where the endemic hot spots are [61, 62].

The *Strongyloides* report developed for this study provides prevalence information relevant to the local health service and community. Increasing the number of persons tested and treated for chronic strongyloidiasis helps to reduce the human reservoir of infection and prevent transmission of infection to others in the community [33]. *Strongyloides stercoralis* has only a limited time in the environment with a maximum of one generation of free-living adults, whereas the autoinfection cycle enables it to live for decades in humans [3, 4, 7, 8, 63, 64]. An on-going testing and treating program in the local health service can identify persons with new or recurrent infections. These persons with recent acquisition of infection can help to identify local sources of environmental or household infection that need an environmental health response to interrupt further transmission.

### Strengths

This pragmatic research design utilised the adult preventive health assessment and the existing electronic patient information and recall system to improve clinical practice by incorporating a systematic process. The prospective, longitudinal nature of the study provided evidence of a sustainable and robust system that was tested in a real-world primary health care setting and able to be repeated in other endemic communities or high-risk populations with similar health service infrastructure.

### Limitations

This study was limited to the role of health services in endemic communities in Australia. In this real-world study, the numbers of current residents aged over 15 years fluctuated thus altering the population denominator at each half-yearly measurement. This same dynamic population denominator was the same as used by clinicians planning other population health activities [52].

This study did not provide universal coverage of the population and was dependent on the settings and other variables including the capacity of the clinical staff to complete AHCs when also delivering acute care to a population with a high burden of disease [52]. This study did not test those under 15 years of age, as collecting blood for chronic diseases is not routine in this age group. Deworming with single dose albendazole for children aged 6 months to 14 years remains routine practice within the preventive child health check in this region [22, 53]. Although non-Indigenous persons have also acquired infections in endemic settings [65], they were not included in this analysis. Therefore, this integrated testing within the AHC can identify undetected strongyloidiasis but needs to be seen as just one important component in the ongoing surveillance and response to *Strongyloides* in these endemic communities.

This study focussed on measuring the change in coverage as a result of implementing the intervention and did not analyse follow-up serology results or reinfection rates. These analyses will be presented in a future publication.

In conclusion, this population health systems intervention successfully increased the coverage and detection of positive cases of chronic strongyloidiasis in four endemically infected communities. Using the electronic patient information and recall system in a primary health care setting, testing was successfully integrated into the Indigenous adult preventive health assessment. The half-yearly *Strongyloides* reports demonstrated the increase in coverage was attributable to the intervention. This sustainable intervention would be beneficial to other endemic communities with similar health infrastructure.

## Supporting information

S1 Table *Strongyloides* point coverage reports. (DOCX)

S2 Table Half-yearly activity measure. (DOCX)
