# Supplementary material for: Integrating testing for chronic strongyloidiasis within the Indigenous adult preventive health assessment system in endemic communities in the Northern Territory, Australia: An intervention study
Source: PLoS Negl Trop Dis. 2020 May 13;14(5):e0008232. doi: 10.1371/journal.pntd.0008232 (PMC7219702; doi:10.1371/journal.pntd.0008232)
Supplement: S1 Table — (DOCX) [file pntd.0008232.s001.docx]

**S1 Table**: Strongyloides Point Coverage Reports extracted at half-yearly intervals (cumulative over the entire 4.5 years study period, starting in July 2012) of persons tested at least once for strongyloidiasis as recorded for the resident population aged 15 years and over by four clinics in remote locations of the Northern Territory, Australia. Number of people = number of current resident adult population at the end-date of the report (dynamic population). N (%) with test = persons tested at least once since July 2012 who were current residents at the time of data extraction. Persons are counted only once in each clinic.

|  | **July 2012 to December 2012** | **July 2012 to June**  **2013** | **July 2012 to December 2013** | **July 2012 to June**  **2014** | **July 2012 to December 2014** | **July 2012 to June**  **2015** | **July 2012 to December 2015** | **July 2012 to June**  **2016** | **July 2012 to December 2016** |
| --- | --- | --- | --- | --- | --- | --- | --- | --- | --- |
| ***Clinic A*** |  |  |  |  |  |  |  |  |  |
| Number of people | 123 | 121 | 128 | 141 | 145 | 160 | 170 | 179 | 195 |
| N (%) with test | 47 (38.2%) | 84 (69.4%) | 100 (78.1%) | 111 (78.7%) | 120 (82.8%) | 132 (82.5%) | 143 (84.1%) | 151 (84.4%) | 164 (84.1%) |
| 95% CI^ | 29.6 to 47.4 | 60.4 to 77.5 | 70.0 to 84.9 | 71.0 to 85.2 | 75.6 to 88.5 | 75.7 to 88.0 | 77.7 to 89.3 | 78.2 to 89.3 | 78.2 to 88.9 |
| ***Clinic B*** |  |  |  |  |  |  |  |  |  |
| Number of people | 217 | 183 | 198 | 234 | 254 | 293 | 343 | 409 | 464 |
| N (%) with test | 69 (31.8%) | 101 (55.2%) | 131 (66.2%) | 152 (65.0%) | 168 (66.1%) | 201 (68.6%) | 235 (68.5%) | 277 (67.7%) | 311 (67.0%) |
| 95% CI^ | 25.7 to 38.4 | 47.7 to 62.5 | 59.1 to 72.7 | 58.5 to 71.1 | 60.0 to 71.9 | 63.0 to 73.9 | 63.3 to 73.4 | 63.0 to 72.2 | 62.5 to 71.3 |
| ***Clinic C*** |  |  |  |  |  |  |  |  |  |
| Number of people | 890 | 556 | 552 | 537 | 552 | 557 | 583 | 624 | 776 |
| N (%) with test | 75 (8.4%) | 122 (21.9%) | 140 (25.4%) | 170 (31.7%) | 218 (39.5%) | 256 (46.0%) | 297 (50.9%) | 342 (54.8%) | 412 (53.1%) |
| 95% CI^ | 6.7 to 10.4 | 18.6 to 25.6 | 21.8 to 29.2 | 27.7 to 35.8 | 35.4 to 43.7 | 41.8 to 50.2 | 46.8 to 55.1 | 50.8 to 58.8 | 49.5 to 56.7 |
| ***Clinic D*** |  |  |  |  |  |  |  |  |  |
| Number of people | 1309 | 1300 | 1225 | 1272 | 1288 | 1305 | 1280 | 1310 | 1375 |
| N (%) with test | 32 (2.4%) | 50 (3.9%) | 78 (6.4%) | 89 (7.0%) | 116 (9.0%) | 263 (20.2%) | 484 (37.8%) | 606 (46.3%) | 724 (52.7%) |
| 95% CI^ | 1.7 to 3.4 | 2.9 to 5.0 | 5.1 to 7.9 | 5.7 to 8.5 | 7.5 to 10.7 | 18.0 to 22.4 | 35.2 to 40.5 | 43.5 to 49.0 | 50.0 to 55.3 |

^95%CI = 95% exact binomial confidence intervals
