# Supplementary material for: Integrating testing for chronic strongyloidiasis within the Indigenous adult preventive health assessment system in endemic communities in the Northern Territory, Australia: An intervention study
Source: PLoS Negl Trop Dis. 2020 May 13;14(5):e0008232. doi: 10.1371/journal.pntd.0008232 (PMC7219702; doi:10.1371/journal.pntd.0008232)
Supplement: S2 Table — (DOCX) [file pntd.0008232.s002.docx]

S2 Table: Half-yearly activity measure of serology testing for strongyloidiasis by four clinics in remote locations of the Northern Territory, Australia. Number of people = resident population aged 15 years and over excluding the persons whose last test had been carried forward in the cumulative half-yearly results table (S1). N (%) with test = number of newly tested persons in each half-yearly interval.

|  | **July to December 2012** | **January to June**  **2013** | **July to December 2013** | **January to June**  **2014** | **July to December 2014** | **January to June**  **2015** | **July to December 2015** | **January to June**  **2016** | **July to December 2016** |
| --- | --- | --- | --- | --- | --- | --- | --- | --- | --- |
| ***Clinic A*** |  |  |  |  |  |  |  |  |  |
| Number of people | 123 | 94 | 75 | 75 | 67 | 85 | 86 | 102 | 102 |
| N (%) with test | 47 (38.2%) | 57 (60.6%) | 47 (62.7%) | 45 (60.0%) | 42 (62.7%) | 57 (67.1%) | 59 (68.6%) | 74 (72.6%) | 71 (69.6%) |
| 95% CI^ | 29.6 to 47.4 | 50.0 to 70.6 | 50.7 to 73.6 | 48.0 to 71.2 | 50.0 to 74.2 | 56.0 to 76.9 | 57.7 to 78.2 | 62.8 to 80.9 | 59.7 to 78.3 |
| ***Clinic B*** |  |  |  |  |  |  |  |  |  |
| Number of people | 217 | 147 | 134 | 144 | 143 | 179 | 219 | 254 | 261 |
| N (%) with test | 69 (31.8%) | 65 (44.2%) | 67 (50.0%) | 62 (43.1%) | 57 (39.9%) | 87 (48.6%) | 111 (50.7%) | 122 (48.0%) | 108 (41.4%) |
| 95% CI^ | 25.7 to 38.4 | 36.0 to 52.6 | 41.2 to 58.8 | 34.8 to 51.6 | 31.8 to 48.4 | 41.1 to 56.2 | 43.9 to 57.5 | 41.8 to 54.4 | 35.3 to 47.6 |
| ***Clinic C*** |  |  |  |  |  |  |  |  |  |
| Number of people | 890 | 509 | 450 | 451 | 443 | 396 | 406 | 421 | 518 |
| N (%) with test | 75 (8.4%) | 75 (14.7%) | 38 (8.4%) | 84 (18.6%) | 109 (24.6%) | 95 (24.0%) | 120 (29.6%) | 139 (33.0%) | 154 (29.7%) |
| 95% CI^ | 6.7 to 10.4 | 11.8 to 18.1 | 6.0 to 11.4 | 15.1 to 22.5 | 20.7 to 28.9 | 19.9 to 28.5 | 25.2 to 34.3 | 28.5 to 37.7 | 25.8 to 33.9 |
| ***Clinic D*** |  |  |  |  |  |  |  |  |  |
| Number of people | 1309 | 1272 | 1181 | 1198 | 1211 | 1219 | 1088 | 923 | 925 |
| N (%) with test | 32 (2.4%) | 22 (1.7%) | 34 (2.9%) | 15 (1.3%) | 39 (3.2%) | 177 (14.5%) | 292 (26.8%) | 219 (23.7%) | 274 (29.6%) |
| 95% CI^ | 1.7 to 3.4 | 1.1 to 2.6 | 2.0 to 4.0 | 0.7 to 2.1 | 2.3 to 4.4 | 12.6 to 16.6 | 24.2 to 29.6 | 21.0 to 26.6 | 26.7 to 32.7 |

^95%CI = 95% exact binomial confidence intervals.
